# Observations on the Reunion of Fractured Bones; with Cases, Illustrating the Utility of Pressure in the Treatment of Ununited Fracture

**Published:** 1828-12

**Authors:** Thomas H. Wright


### REUNION OF FRACTURED BONES.

*Observations on the Reunion of Fractured Bones; with Cases, illustrating the Utility of Pressure in the Treatment of Ununited Fracture.* By THOMAS H. WRIGHT, M.D.

IT is not the purpose of the present communication to investigate particularly the causes which obstruct or defeat the efforts of the constitution to restore the unity of fractured bones. Those causes are various, and perhaps are generally understood, though few attempts seem to have been made to elucidate their nature, or point out their mode of influence. The main impediment to the process in question has been referred, in general terms, to debility, or a certain defect of power, either of the whole system or of the part, to set up and maintain the proper actions. This state of disability may be the effect of causes which, either by their intensity or continuance, may depress the powers of the system below the point necessary to the repair of great local injuries; or some specific causes, affecting the power of the constitution to preserve or restore its integrity of structure, are supposed to display their character and effect chiefly in suspending or vitiating the action of ossification. Among the latter are ranked the severer forms of scrofula, syphilis, scurvy, &c.

Whatever may be the effect of general or special causes on the constitution in interrupting or altering the actions of nutrition, and particularly of the nutrient office of the arteries of bones, it seems probable that the state of ununited fracture, as it is commonly found, is often the result of circumstances independent of any remarkable general or specific disability of the whole system. Such a consequence is, perhaps, usually induced by causes whose operation or effect is confined very much, if not altogether, to the point of injury.

It is to local circumstances, either arising out of the character of the lesion, or casually interfering with the design and efforts of the constitution to repair the injury, that we are to look for the ordinary causes of ununited fracture. To the first class belong bad forms of compound or complicated fracture, in which there arises a necessity for extensive exfoliations, attended by inflammation, and perhaps repeated and profuse suppuration, before the bones can be brought into a state to undertake the work of permanent reunion. Here the energies of the parts become subdued by their being kept so long in a state of irritation, and forced into the performance of actions alien to restoration.

Another class of causes which tend to frustrate the design of nature in the repair of fractures, and the operation of which seems to be purely local, are all the circumstances which prevent such relation and repose of the parts as are essential to the perfection of the work to be performed. A certain correspondence of surfaces, and concert of action between the fractured extremities, are indispensable to firm union; and whatever tends to obstruct, either constantly or frequently, the state of contact and rest, will commonly hinder, in part or altogether, the act of reunion. Hence imperfect reduction or apposition of fractures, errors of position, improper or inefficient means of support, frequent or causeless movement of the parts, unrestrained mobility of the patient, inattention to the state and effect of the supporting means, the too early removal of the necessary defence by splints and bandage, and, lastly, premature or untimely attempts to bring the parts into action and use: all these, and more of a similar tendency, are calculated to disturb, and at last to defeat a process which can only be perfected when wholly guarded from such interruptions.

I readily assent to the general propriety with which the advanced and declining epochs of life may be considered as unpropitious to the active functions of growth, nourishment, and renovation of parts, yet I have been frequently struck with the resources of the system in old age, as displayed in the prompt and perfect repair of injuries both of the soft and solid parts of the body. In the closing of wounds, the filling up and healing of extensive ulcers, and the firm reunion of fracture, it has occurred to me to observe all those processes accomplished with a facility and completeness scarcely exceeded at any age, in some instances where the subjects of such accidents had passed the eightieth year of life.

Ossification, or calcareous conversion of the vascular tunics, though a common occurrence at that period, is not the invariable concomitant of senescence; and, though seldom occurring before middle age is past, does not correspond, either in origin or degree, to the relative advance in life of the individuals in whom such change may exist. It is found to have occurred in some persons at forty or fifty, in others at sixty or eighty years, and is sometimes present in a greater degree, or more manifestly, at the former than at the latter age. From all the means of judging which a few examples afford, I am inclined to the opinion that some degree of ossific degeneration of the vessels of the extremities would not invariably or necessarily involve a failure of bony union, should fracture occur to limbs thus circumstanced. It has been my duty to attend to more than one case of fracture of the leg in individuals exhibiting distinctly free calcareous conversion of the tunics of the arteries of both the upper and lower extremities, where they were accessible to examination. Those cases presented ultimate apparent* consolidation of the fracture, though the process of bony union was sometime doubtful and of tardy accomplishment, requiring unusual confinement, and more than common attention to the sustaining and intimate relation of parts. Possibly in those cases where bony union obtains in fractures, notwithstanding a calcareous state of the vessels of the limb, the nutrient arteries of the bones may not be affected by the change which the capillary series of the surrounding soft parts have undergone.

The following cases are reported to illustrate a principle in the treatment of ununited fracture, which the untoward and embarrassing circumstances of the first case suggested, as offering the best promise of averting the great evil of permanent disability from such an accident. At the time this case was under treatment, I had met with no account of the similar intention, first acted upon, it would seem, by Mr. Amesbury, and since practised in one instance by Mr. Brodie, and possibly by others. I allude to *pressure*, applied with the design of maintaining ununited fractured surfaces in a state of more strict and continued apposition and contact than had been accomplished or attempted by the means usually employed.

In the excellent Treatise on Fractures and Dislocations presented to the profession by Sir Astley Cooper, the subject of pressure in fractures of tardy union is briefly

* I use the term apparent, because the absence of obvious or sensible motion at the point of former fracture is not absolute proof of bony consolidation. The point of fracture is sometimes so braced by ligamentous connexion that no ordinary force will cause obvious movement in the part.

noticed; though it seems to have been resorted to chiefly with the design of maintaining apposition in cases where, from the nature and seat of the injury, there was particular tendency to separation of the fractured surfaces.* The work of Sir A. did not come into my hands until some time after the termination of the first case reported, in which the expedient of pressure was suggested by the necessities of that case, and successfully employed.

Cases of false articulation, as they were formerly termed, have long been subjects of interest and embarrassment to surgeons, and were for the most part unmanageable by the resources of art employed for their correction. It was early discovered that the cause of the defect consisted in a failure of the ossific process, and the substitution of a species of ligamento-cartilaginous matter, by which the fractured ends of the bone were covered, and separately rounded, so as to permit them to glide or move freely upon each other. The obvious indication suggested by this state of parts in ununited fracture was to excite a new action in the fractured extremities, by which the natural bond of union might be produced. Hence we find the first attempts to correct false joints were regulated by the intention of creating a state of inflammation in the ends of the bone, thus stimulating them to throw out the matter necessary to their consolidation. The earliest mean by which the design was attempted to be accomplished was friction of the points of fracture upon each other, as frequently and forcibly as was deemed necessary to insure a new state and new action in the parts. This method was said to have sometimes succeeded in establishing solid union; but the plan seems to have been loosely conducted, was soon in a great degree laid aside, and perhaps never effected what it intended. One prominent defect of the scheme was, that no means appear to have been adopted to maintain such apposition of parts, subsequent to the frictions, as was necessary to facilitate reunion.

A bolder scheme of treatment in ununited fracture succeeded. It consisted in cutting down to the false articulation, and sawing off the smooth extremities of the bone, thus reducing the parts as nearly as possible to the state in which they had been at the first moment of injury, and treating the case *de novo* as a compound fracture. This plan has been often tried, with various success and controverted credit. The French surgeons (Boyer and others) say it is an expedient

* See Case of Fracture near the Condyles of the Femur, reported by Mr. Wellbank, page 180.

seldom fortunate, and practicable only on limbs with single bones, the femur and humerus. The English surgeons (White, Wardrop, &c.) speak confidently in commendation of this adventurous experiment, and of its successful application to double as well as single bones. The plan in question has certainly been sometimes effectual, but the serious degree of both local and constitutional irritation, which has often ensued in this operation, is justly considered a formidable objection to its employment, and it is now seldom practised.

An important improvement on the plan of treating ununited fracture first practised, was suggested and brought into use about the year 1804, by Mr. White, of Manchester. Acting on the intention originally consulted, he at the same time placed the parts under circumstances favorable to the end proposed, and thus remedied the defect which had almost always frustrated the design under the first method of treatment. Mr. White applied to the limb in which a false joint existed (the thigh, for example,) a strong case, made of sole or saddle leather, lined with a soft material, and adapted to the length and figure of the limb. The case extended from the great trochanter, ileum, and ramus of the pubis, to the patella and condyles, was made to embrace the thigh as closely as possible, and buckled firmly on by straps, so as to prevent the ends of bone at the false joint from sliding out of their proper and direct apposition to each other. The case was worn constantly for the necessary period, was kept firmly braced by the straps, and the patient directed to walk about, and use the limb as much as possible. Drs. Inglis and Brown report cases of false articulation treated in the Royal Infirmary of Edinburgh on the plan of Mr. White, with complete success. Examples are related of patients in the Infirmary with ununited fracture of the thigh, upon whom the case was applied, the patient sent into the country, with the proper directions for the management of the apparatus, and his returning in a month or two afterwards, walking well and firmly, and bringing the case in his hand to restore it to the institution. The period of time for which it was found necessary to maintain strict support by the case and straps, varied from five weeks to two or three months.

It is evident that the design and management by the case and straps, with free motion of the limb, as just described, differs somewhat from that by pressure, with rest, or what may be termed still-pressure. In the one case, the direct gravitation of the body upon the false articulation seems calculated to induce a high degree of irritation upon the antagonising points of bone, while the very free use of the member in locomotion must cause a constant, though limited, range of friction between the relative surfaces. Such a state of things, namely, great pressure and constant play of parts, would appear likely to produce bad consequences in one of two ways, either by inducing excessive local and constitutional irritation, or, on the other hand, by accustoming the parts to forcible contact and free movement, to confirm the state of insensibility and indolence of action which had caused the original failure of union. But adversary hypothesis must be silent in the presence of facts. We are told, on good authority, that the plan has been often and completely successful. By the process of pressure with rest of parts, nothing more is designed than first to promote absorption of the foreign covering of the fractured points, and thus attaining and preserving an intimate and uniform approximation of bony surfaces. If this can be attained, the system and the laws of the part will generally accomplish the rest. Without speculation on the why or wherefore of such actions, we know that if the divided surfaces of bone can be brought and maintained in a state of closeness, without the intervention of any substance foreign to their own constitution, they possess, and will exert, a natural tendency to unite by bone; or, what is the same thing, actions of formation take place under such circumstances, which actions, when perfected, result in a solid homogeneous contexture of parts.

Case I.—A master carpenter, ascending to the roof of a house, (October 1826,) slipped from the ladder when near the top, and fell about twenty feet to a scaffold raised a small height above the pavement. He dropped to the scaffold so that the momentum of the body and the force of concussion was sustained chiefly on the right limb, which was fractured just above the ankle. The fracture was a very bad one, traversing the tibia obliquely for some inches, and rendered compound and complicated by fracture of the fibula also, and the penetration of the superior fragment of the tibia through a ragged wound of the integuments, two inches in extent. The patient was seen in a few minutes by a physician of knowledge and experience, by whom the parts were promptly adjusted in the best possible manner. After the patient had been conveyed home, the interest and alarm of some of his friends induced them to urge calling myself also to the case. I accordingly saw the patient in concert with the gentleman who attended in the first instance.

On examining the injury, the fractured points were found fairly together, and requiring no interference; but hemorrhage from the wound was considerable, and, after fruitless attempts to restrain it effectually by compression, it became necessary to seek out and tie one or two (the internal malleolar) arteries. Even then troublesome oozing continued, and as the stream was found flowing from deep in the fissure of the bone, it could only be controlled by putting down on the bleeding point a compress of sponge, which, being pressed in firmly by a probe, put a stop to the bleeding. Compression in this manner was rendered easy in consequence of some vacant space caused by the removal of several pieces of bone which had been found lying loose in the course of the fracture.

The injured limb was extended on a firm surface, and well supported by broad splints on each side, braced above the knee, and reaching some inches below the foot, where the ends of the splints were locked at the proper distance by a transverse bar received into corresponding mortices, and the leg well cushioned within the splints by soft pads made for the purpose. The wound was lightly dressed, and no bandage thrown immediately around the limb. From the lacerated state of the integuments, some comminution of the bones, and the great obliquity of the fracture rendering the point of the upper fragment of the tibia extremely thin, sharp, and probably destitute of sufficient connexions for its support, it was to be expected that high inflammation, and some exfoliation, were likely to ensue. The limb was therefore left without close dressing, to avoid irritation, and that it might be conveniently accessible for such local application as might be deemed necessary, in correspondence with the general treatment, to avert or limit the consequences to be apprehended.

In the course of the second day from the accident, great constitutional disturbance, with irritative fever, sat in, and wore for a short time a menacing aspect. Timely bleeding and purging, followed by a full anodyne impression, allayed the tumult, and brought about a calm and comfortable state.

On the fourth day the wound was examined, the sponge compress removed, and a light dressing applied. The leg was not sensibly affected by swelling or inflammation, and it continued free from any unpleasant state for two weeks from the time of the accident, the wound suppurating well, and the patient's health as sound as usual. At the end of that period a change for the worse occurred. The wound, which had contracted a good deal, became gleety, the integuments tumid and dark coloured; the limb swollen, hot, and painful; the patient was at the same time affected by chills and flushing. This state of things ended in abscess, re-opening of the wound, and exfoliation from the point of the upper fragment. It is unnecessary to encumber the narrative with a minute account of the treatment pursued. The antiphlogistic and soothing plan, general and local, were steadily enforced, as far as circumstances indicated.

After this interruption, circumstances again wore a favorable aspect, and by the 28th of November (six weeks after the accident,) the limb had lost all remains of inflammation and swelling; the wound was almost closed, a good deal of apparent firmness about the fracture, and there seemed every probability of perfect consolidation. This pleasing expectation was speedily disappointed. Inflammation and tumefaction again took place, followed by suppuration, and more pieces of bone were found detached, and taken away. During this second painful process of suppuration and exfoliation, the limb lost all that was previously accomplished toward reunion ; and now, at the end of eight weeks, the parts could not be regarded as more advanced towards recovery than on the first day of the injury. The fractured ends of the tibia moved free and loose, and an open wound of some extent communicated with the chasm or fissure between the fractured points.

The consequences of this second suppurating and exfoliating action gradually passed off, and again there seemed an almost successful effort on the part of the constitution to restore the entireness of both the solid and soft parts. The wound closed in, the bone became comparatively firm, and the limb recovered nearly its natural appearance.

Such continued the state of the parts for about two weeks succeeding the last occurrence of suppuration, &c.; in all, ten weeks from the date of fracture. Then ensued a condition of parts more inauspicious than ever. Inflammatory œdema supervened, the whole limb swelled to great bulk, vesications formed every where on the surface, and the characters of œdematose and vesicular erysipelas were displayed over the limb in almost their worst form. This new and threatening state of things called for very great care, to prevent consequences serious alike to the part and to the system. Mild fomentations diligently maintained, strict repose of the parts, and soothing general means, aided by a constitution naturally excellent, and never abused, fortunately led on to the least hurtful termination which circumstances admitted. Free suppuration took place in the limb, the matter was discharged by incision, and the local and constitutional irritation gradually subsided. No exfoliation followed this greater than either the preceding suppurations, but there had now arrived a crisis truly discouraging. The whole business of repair was to be commenced anew for the third time. Every mark of bony union had disappeared under the high inflammation, great tumefaction, and suppuration, which severely tried the life and organization of the whole limb. The new formations, being least able to bear the change, had all given way; and, when the tension of the parts relaxed, the bones were found lying without connexion, the lower fragment, with the foot attached, yielding in any direction to the slightest impulse, and requiring the most careful support to prevent the foot from declining, by its own weight, very much out of the proper axis of the limb.

Nearly three months had passed since the date of the fracture, and nothing effectual had been done toward the important end of repairing the broken bone.* But there was still room to hope for ultimate success. The patient's constitutional powers remained firm in a great degree under so many depressing and disturbing causes. Though affected considerably during the acute periods of inflammation and suppuration in the limb, his system rallied promptly and fully when these states passed by. Strict repose of the part was still enforced, the limb kept in position, sustained by external splints and an interior padding, adapted to all the curvatures of the leg. By perseverance in these measures, the limb put on a state of great improvement, the natural figure and size was regained, and the tendency to inflammation seemed to be entirely lost. In three weeks after the last-mentioned suppuration, (fourteen from the accident,) the wound of the integuments was healed, and the whole exterior of the limb looked in every respect well.

The splints were now removed, to ascertain by a cautious examination the state of the bone in the line of fracture. We had then the mortification to discover a total failure in the main object of all our care. There existed a manifest disjunction of the tibia throughout the whole extent of fracture; and so complete was the disunion, that, when the leg rested on the hands above the point of fracture, the foot dropped away from the upper fragment, causing the point of the latter to project one-fourth of an inch above the level of the portion of the tibia attached to the foot. By the gravitation of the foot and inferior fragment, the line of the fissure was also made to open so sensibly that the point of the finger could be insinuated in some degree between the sides of the fracture, carrying the integument before it into the chasm. Rotation of the foot likewise caused an obvious play of alternate opening and closing in the line of separation.

Not wholly discouraged by this unpromising state, it was determined to persevere in such measures as seemed suited to promote final union. A close bandage, hitherto inapplicable from tenderness and proclivity to inflammation, was applied moderately tight, to give more direct support above the fracture, and the external splints continued. Under this treatment the limb remained free from inflammation, swelling, or pain; the wound of the soft parts was firmly repaired; and the colour, shape, and feeling of the limb the same as before the accident. But raising the leg from the splints, or rotating the limb by the foot, it was evident that the tibia was totally discontinuous at the point of fracture. The fractured surfaces could be brought together, and made to move liberally on each other; but there was no longer any sensible crepitation. The inequalities proper to fracture were filled in, and smoothed over, by that fibrous deposit which comes at last to constitute the covering of fractured surfaces that have missed the

* This remark applies to the tibia. The fibula had united early, and did not afterwards give way.

bony reunion. The prospect of ultimate consolidation by the energies of the constitution, and the common mode of local management was utterly extinguished in the present case, by the state of the limb as just represented.

While anxiously looking for some resort calculated to avert from a worthy man, with a large family, the terrible evil of permanent lameness, it occurred to us that we might find in pressure, steadily maintained, the mean best suited to the indication in the present case. It was plain that mere apposition was not sufficient, under the peculiar circumstances of the case, to induce consolidation. The parts had been kept in a state of approximation and repose for four weeks succeeding the last suppuration, yet no bony union resulted. Something more than mere propinquity and rest of parts was now required. It had become necessary to cause absorption of the substance formed on the ends of the fracture, and at the same time to excite on those surfaces new attempts to the deposition of bone. Pressure presented the best promise of effecting those objects; and, to be efficient, it was evident that it must be as strict as the health of the soft parts would permit, and continued without remission for a sufficient time.

The simple, efficient, and surgical mode of making pressure by the tourniquet, since successfully practised by Mr. Brodie, did not occur to me. A strong, soft, double napkin was passed under the leg, extending from below the ankle to the head of the tibia; pad compresses of suitable size were laid, which, when braced by the general bandage, acted as counter-wedges on the line of fracture. The bandage or napkin first laid was drawn firmly around the limb, and confined at short spaces, at first by large pins, and afterwards by tacks. It was thus made to give uniform support and a regulated pressure through the compresses on the line of fracture, and to adapt itself closely to the contour of the whole limb from below the ankle, sustaining the inferior fragment to the top of the leg. The principle, in short, was that of the laced stocking, modified to act especially on a definite point and space. The compressing bandage was inspected, and generally tightened, twice a day, and always drawn as firmly as the patient's feelings would allow. The limb was kept extended and in splints, to maintain steadiness, and preserve the correct axis of the foot on the leg.

The favorable tendency of this treatment soon became manifest. In ten days the chasm or interval of the edges of the bone along the line of fracture was much diminished, the bones having sensibly approximated by absorption of the interposed deposit. After three weeks the depression which had always marked the line of fracture, and extent of separation, was obliterated to the touch, and the ends of bone seemed in close contact. Now, too, the tendency to eversion of the foot was lost, and moderate lateral pressure on the foot did not cause any parting at the fissure.

A further particular detail is unnecessary: perseverance in the course described produced, at the end of five weeks from its adoption, unequivocal bony union. At that period the patient could elevate the whole limb; flex, extend, and rotate the foot freely, without the slightest evidence of motion in the line of fracture. The splints were thrown off, but the compressing bandage directed to be worn less firmly applied for some weeks, and the patient admonished to avoid for the present throwing the weight of the body on the affected limb.

The cure in this case became perfect, and when the limb was enough recovered to take its share of weight and motion, it was found to have shortened less than one-fourth of an inch. The man walks now without halting, and as firmly and well on one limb as the other. This patient was confined to bed nineteen weeks and two days; and it was six months from the accident before the consolidation was sufficient to permit use of the limb in walking.

Case II.—In August 1827, a man, aged sixty-one years, designing to elope from Baltimore Almshouse, to get liquor, jumped or dropped from a wall twelve feet high. He alighted on his feet, but immediately fell, and was unable to rise again, so as to stand or walk. He was soon discovered, and brought into the surgical ward, when, on examination, the left leg was found broken, both bones, near the articulation with the foot. The fracture of the tibia was very oblique and almost compound, the integument being partially cut through by the projecting upper fragment.

The bones were adjusted, the limb moderately extended and laid in a fracture-box well cushioned, but was left without close bandage, to admit the free use of applications designed to allay inflammation and swelling, which had commenced, and were likely to be severe. Very great intumescence followed, with diffused inflammation, pain, &c., and it was nearly a fortnight before these were reduced sufficiently to allow close dressing. The limb was then braced around by an eighteen-tailed bandage, and sustained in position by the leg-splints of Sir A. Cooper, formed with the lateral supports for the foot. The patient's constitution stood the shock without disturbance, and every thing went on apparently well for five weeks, the bandage and splints being adjusted as occasion required. At the end of that period the splints and bandage were removed, to examine the state of the fracture. The fibula was found consolidated, but there was free motion at the line of fracture through the tibia. No crepitation could be distinguished, though the fractured surfaces were made to glide on each other with considerable force. The eighteen-tailed bandage was again wrapped closely about the leg, the limb placed in position, and the splints reapplied. This plan was continued three weeks longer, (eight weeks entire,) when the fracture of the tibia was found still ununited.

The case was altogether unpromising. The patient was above sixty years of age, and, although shewing the remains of a naturally good constitution, was, in many respects, a bad subject for fracture. Previous to the accident, the leg was in a state of chronic enlargement, from infiltration and thickening of the cellular tissue. On the leg not fractured there existed a large ulcer, of some years duration, which could not, by the utmost attention, be brought to cicatrise. Superadded to these evidences of diminished power of natural action in the lower limbs, this patient shewed, in all the tangible arteries of the extremities, an advanced degree of calcareous transformation. An indurated and knotty state of the tunics was distinctly traceable in all the superficial trunks; and it is then always probable that similar degeneration obtains in the ultimate vascular series of nutrition.

It was determined to try strict and continued pressure. With this intention, a roller was laid regularly from the toes to the knee. Over this was applied short splints, made of binder's boards cut to suit the outline of the limb, then macerated, that they might be made to adapt themselves intimately to the surface about the fracture. These splints were laid, while pliable from wetness, on opposite sides of the fracture, and braced in their place, as firmly as could be borne, by a second roller, carried from the ankle to the top of the leg. Two lateral splints of the same material, not macerated, were next applied, and secured at intervals by a few turns of a roller. These splints were sufficiently long to steady the leg and prevent motion of the foot; the wood splints first used were laid aside, and the patient confined to bed, with the limb extended. An occasional change from the recumbent to the sitting posture was allowed the patient; but he was altogether prohibited from getting out of bed or bringing the limb into use.

An evident change in the relation and aspect of the parts was observed in a fortnight after this course was instituted. When the plan of compression was adopted, the superior fragment of the tibia projected so sensibly at the point of separation as to cause much deformity by its remarkable prominence. At the end of two weeks this projection was much reduced, and by the fourth week the bone had regained its natural level: doubtless by the influence of pressure on the deposited matter, which had occupied the interval between the fractured surfaces. The facility and range of motion at the fracture were also lessened, though still discoverable. The pressure, together with confinement to position, were steadily maintained for six weeks. The long splints were then taken off, and the patient permitted to put the limb to the floor, and, supported by crutches, to bring it cautiously into use. The roller and close splints were kept on some time longer.

When the point of fracture was examined some weeks after the patient began to use the limb, the part appeared very firm. No pressure, which it was thought proper or necessary to employ, whether directed immediately to the fracture, or by using the foot as a lever to rotate the limb freely, caused the slightest indication of disunion. In a month or six weeks after the limb had been brought into use, the patient threw aside all artificial supports, and walked as well as before the accident. There is no discoverable shortening of the leg.

I have doubts as to the fact of proper bony union in this case; and those doubts rest on the general circumstances of the patient, stated in the narrative as having been adverse to union in the first instance. There is certainly nothing in the state of the part, viewed abstractedly, to warrant the suspicion of heterogeneous connexion ; no force, that can be properly employed, causes motion of the part, nor does it yield at all to the gravitation of the body. But instances have occurred in which pseudo union, or fibrous anchylosis, after fracture, has been so strict and firm as not to have been manifest either by sensible motion or defect of power in the part, and were only casually discovered during some autopsic examination. Even if it be the fact that actual consolidation has not obtained in the case just reported, pressure has not, on that account, been the less an important or beneficial mean of treatment. It has restored the limb to a state of usefulness it would not otherwise have regained. Neither always, nor generally does it happen that the weakness and discomfort of a false joint are spontaneously overcome in course of time. Cases have been shewn to me of disunion of the tibia from fracture, in which after three years the mobility was stated to be greater than it had been at the end of three months, and would have altogether prevented walking but for the partial support of the fibula.

Case III.—A man was brought to the Baltimore Almshouse, 6th of January, 1828, with his head much cut, and his left arm in a state of excessive tumefaction, from blows given with a heavy stick. On examination, the ulna was found broken about the middle, but the inflammation and swelling were so great as to prevent the immediate use of bandage and splints. The limb was kept at rest, under the use of local applications designed to subdue inflammation ; and this being sufficiently accomplished in six or eight days, the points of fracture were coaptated, and well sustained by splints in the usual mode. The forearm was suspended in a state of easy flexion.

Every necessary attention was paid throughout the treatment of this case to preserve proper relation of the bones to each other, repose of the parts, and due support; yet at the end of five weeks the fracture was found as susceptible of motion as at first. This man was at the middle age of life, of apparently sound constitution, and the failure of union could only be accounted for from the very great inflammation, intumescence, and effusion, which had pervaded the limb in consequence of the blows, and the general disability in which the parts had been left when these passed off.

Pressure was resorted to in this instance also. A roller was laid smoothly from the fingers to and some distance above the elbow. Short splints, of binder's board, were fitted to the interior and external surface of the limb, to afford sufficient lateral support, and were well secured in place by a roller. A long splint of binder's board, incurved and adapted to the arm, was laid along its ulnar line, extending below the wrist to support the hand; and a shorter splint of the same material rested on the radial side, the two forming an external case for the arm, not quite close or meeting at the edges. The splints were made to embrace the arm by straps at short spaces, which, particularly at and near the fracture, were drawn as tight as could be borne. A due degree of pressure was thus constantly maintained as long as necessary, and the result was satisfactory in this instance as in the preceding cases. But considering the age and health of the patient, and the character and seat of the fracture, consolidation was here very tardily effected. It required two months' perseverance in the treatment by pressure before motion at the point of fracture ceased altogether. Now, however, fourteen weeks from the date of injury, union is perfect. The slow repair, even under close pressure and strict rest of parts, is perhaps attributable to the same cause which is supposed to have defeated union by the first process, namely, the feeble manner in which the actions of life were performed in the limb, in consequence of its having recently suffered so great inflammatory disturbance.*

* American Journal of the Medical Sciences.

